# Corrigendum to “A survey of pathways for mechano-electric coupling in the atria” [Prog. Biophys. Mol. Biol. 159 (January 2021) 136–145]

**DOI:** 10.1016/j.pbiomolbio.2022.01.003

**Published:** 2022

**Authors:** Marta Varela, Aditi Roy, Jack Lee

**Affiliations:** a National Heart and Lung Institute, Faculty of Medicine, Imperial College London, London, UK; b Department of Biomedical Engineering, School of Biomedical Engineering & Imaging Sciences, King's College London, London, UK; c Department of Computing, University of Oxford, Oxford, UK

The authors regret that the article included the wrong image in Fig. 5. This has now been amended.

**Figure:**
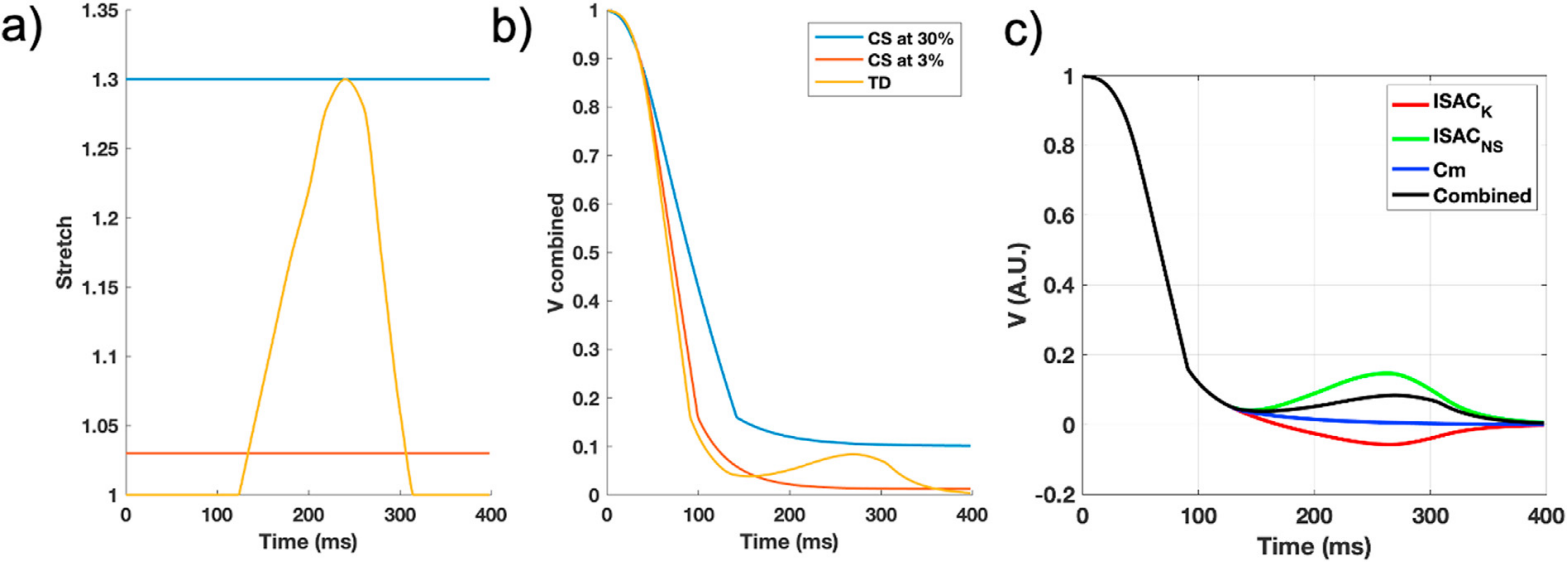

The authors would like to apologise for any inconvenience caused.

